# Hot stuff in the bushes: Thermal imagers and the detection of burrows in vegetated sites

**DOI:** 10.1002/ece3.7491

**Published:** 2021-04-01

**Authors:** Tarnya E. Cox, Robert Matthews, Grant Halverson, Stephen Morris

**Affiliations:** ^1^ Vertebrate Pest Research Unit New South Wales Department of Primary Industries Orange NSW Australia; ^2^ Heli Surveys Jindabyne Airport Jindabyne NSW Australia; ^3^ Airborne Technologies Australia Camden Airport Cobitty NSW Australia; ^4^ Research and Business Excellence New South Wales Department of Primary Industries Wollongbar NSW Australia

**Keywords:** drone, pest animals, remote detection, survey, thermal imager, UAV, warrens

## Abstract

1. Thermal imaging technology is a developing field in wildlife management. Most thermal imaging work in wildlife science has been limited to larger ungulates and surface‐dwelling mammals. Little work has been undertaken on the use of thermal imagers to detect fossorial animals and/or their burrows. Survey methods such as white‐light spotlighting can fail to detect the presence of burrows (and therefore the animals within), particularly in areas where vegetation obscures burrows. Thermal imagers offer an opportunity to detect the radiant heat from these burrows, and therefore the presence of the animal, particularly in vegetated areas. Thermal imaging technology has become increasingly available through the provision of smaller, more cost‐effective units. Their integration with drone technology provides opportunities for researchers and land managers to utilize this technology in their research/management practices.
2. We investigated the ability of both consumer (<AUD$20,000) and professional imagers (>AUD$65,000) mounted on drones to detect rabbit burrows (warrens) and entrances in the landscape as compared to visual assessment.
3. Thermal imagery and visual inspection detected active rabbit warrens when vegetation was scarce. The presence of vegetation was a significant factor in detecting entrances (*p* < .001, *α* = 0.05). The consumer imager did not detect as many warren entrances as either the professional imager or visual inspection (*p* = .009, *α* = 0.05). Active warren entrances obscured by vegetation could not be accurately identified on exported imagery from the consumer imager and several false‐positive detections occurred when reviewing this footage.
4. We suggest that the exportable frame rate (Hz) was the key factor in image quality and subsequent false‐positive detections. This feature should be considered when selecting imagers and suggest that a minimum export rate of 30 Hz is required. Thermal imagers are a useful additional tool to aid in identification of entrances for active warrens and professional imagers detected more warrens and entrances than either consumer imagers or visual inspection.

## Graphical abstract

Thermal imaging technology is a developing field in wildlife management. We investigated the ability of both consumer and professional imagers mounted on drones to detect rabbit burrows (warrens) and entrances in the landscape as compared to visual assessment. Exportable frame rate (Hz) was the key factor in image quality and subsequent false‐positive detections. Thermal imaging technology provides an efficient method for detecting rabbit warrens and entrances in all vegetation types (open, vegetated, and mixed), surpassing visual inspection alone.

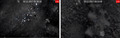

## 1 INTRODUCTION

Since its development, thermal imaging technology has been used in a wide range of applications (see (Vollmer & Möllmann, 2018) for a full review of the use of thermal imaging across study areas). Thermal imaging technologies have been increasingly used in wildlife surveys from the 1960s (see (Croon et al., 1968; Parker & Driscoll, 1972)) although their widespread use has been limited due to the cost of the equipment and a lack of exposure of this type of equipment to biologists. The majority of wildlife survey work undertaken with thermal technology was in the detection of large wild animals such as pigs and ungulates using fixed or rotary‐wing aerial survey techniques (and occasionally comparing these results to visual surveys over the same area) (Focardi et al., 2001; Havens & Sharp, 1998; Parker & Driscoll, 1972). More recently, thermal surveys have been undertaken on larger surface‐dwelling or arboreal species (Corcoran et al., 2019; Spaan et al., 2019). Limited work has been done on abundance estimates of smaller animals and the detection of fossorial animals and/or their burrows with thermal imagers (Boonstra et al., 1994). Burrows of fossorial animals can be difficult to detect during ground surveys, particularly where vegetation is present. Additionally, those burrows that can be found often give little and subjective indication as to whether these burrows are occupied.

The detection of occupied burrows is particularly important when the burrowing animal is a pest, such as rabbits (*Oryctolagus cuniculus*) in Australia. Rabbits are a significant agricultural and environmental pest and are listed as a key threatening process (Commonwealth of Australia, 2016). Rabbits have been estimated to cause > AUD$206 million per annum in agricultural losses (Gong et al., 2009). Additionally, it was estimated that private and public landholders spend approximately AUD$6 million per annum controlling rabbits. They are listed as a direct threat for 321 species of Australian plants and animals and 75 endangered ecological communities (Commonwealth of Australia, 2016). The most effective long‐term method of controlling rabbits is the removal of their harbor. Usually, this means the destruction of their burrow (hereafter referred to as a warren) through ripping programs; however, the success of ripping programs is greatly influenced by the presence of surrounding active warrens (McPhee & Butler, 2010). Reopening of ripped warrens can occur if any nearby warrens remain intact; therefore, it is essential that all warrens and warren entrances within the treatment area are located. Where rabbit numbers are high (>5 rabbits/Ha), warrens can be easy to locate due to the lack of vegetative cover. However, where numbers are lower, or nonpalatable plants are abundant, warren entrances can be obscured and difficult to find.

Thermal imagers may provide a way to detect these obscured rabbit warrens. Boonstra et al. (1994) used thermal imagers to differentiate between occupied and unoccupied arctic ground squirrel (*Urocitellus parryii*) burrows (where the location of the burrow was known). They also identified that the presence of dense vegetation was a limiting factor in thermal surveys. Technological development of thermal imagers has progressed rapidly over the last 10 years, and there is a proliferation of thermal imagers available for consumers. Therefore, it was time to re‐evaluate thermal imagers for the detection of animal burrows. We investigated whether consumer thermal imagers could be used as a tool to assist land managers with identifying rabbit warrens, particularly if obscured by vegetation. Here, we investigate the use of thermal imagers to (a) determine whether active and inactive rabbit warrens could be detected with a thermal imager and (if so) (b) to evaluate the efficacy of consumer imagers to professional imagers and visual inspection.

## 2 MATERIALS AND METHODS

### 2.1 Site locations

The evaluation took place in two parts over three properties in New South Wales, Australia. Part 1 was undertaken on two private properties in the Central Tablelands in November 2017. Part 2 occurred on public land in the Central West in June 2018. We classified the rabbit populations at each of these locations as very high (>10 rabbits/Ha), high (>5 rabbits/Ha), medium (2–5 rabbits/Ha), and low (<2 rabbits/Ha) using standard white‐light spotlight counts (Mitchell and Balogh 2007). For Part 1, one property was overgrazed and had little‐to‐no vegetation and a very high population of rabbits. The other property was a disused stock paddock that contained stands of blackberry (*Rubus fruticosus* species aggregate) on the gully lines and was overrun with serrated tussock (*Nassella trichotoma*). This property had a low rabbit population. Rabbit warrens on these two properties had an average depth of 600–800 mm (based on information from previous excavation and control programs) and we surveyed five warrens at each property. For Part 2, the land was part of the national traveling stock route (authorized thoroughfare for the walking of domestic livestock from one location to another across Australia) and at the time of the survey consisted of open sandy country with stands of Old Man Saltbush (*Atriplex nummularia*). The average rabbit warren depth was 1,500–2,500 mm (based on information from previous excavation and control programs), and the rabbit population was classified as very high. We surveyed a 6.11 Ha portion of the area. Warren depths were determined through excavation undertaken during previous control programs.

### 2.2 Equipment used

We used three uncooled microbolometer arrays (Table 1) of varying sensor size and cost. The Jenoptik VarioCAM^®^ HD (hereafter referred to as the “Jenoptik”) professional thermal imager was used to evaluate part 1, with the FLIR Zenmuse XT640 and Sierra‐Olympic VayuHD used in part 2 (hereafter referred to as the “Zenmuse” and “Vayu,” respectively). The Zenmuse came as an integrated system with the DJI Inspire 1 drone; however, both the Jenoptik and the Vayu were heavier nonintegrated imagers. Both of these imagers required mounting to a Ronin MX gimbal (https://www.dji.com/au/ronin‐mx) for image stabilization. The Jenoptik was mounted to a DJI S1000 + drone (https://www.dji.com/au/spreading‐wings‐s1000/spec) and the Vayu mounted to a DJI Matrice 600 drone (https://www.dji.com/au/matrice600/info#specs). All video was collected and processed as “white‐hot” grayscale imagery. The UAV Operator held a Remote Operators Certificate (ReOC) for the appropriate weight class of the UAV and with an instrument (Exemption from the regulations issued by the Civil Aviation Safety Authority) to operate at night. The pilot was qualified and approved for night operations under the operator's ReOC Operations Manual.

**TABLE 1:** The three thermal imagers (uncooled microbolometer arrays) used during the study

Imager	Drone	Hz (view)	Hz (export)	Sensor (w × h) mm	Image (w × h) px	Pixel pitch	Cost ($AUD)
FLIR Zenmuse XT 640	DJI Inspire 1	30	<9	12.38 × 9.68	640 × 512	17 μm	~AUD$20K (integrated)
Jenoptik VarioCAM^®^ HD	DJI S1000+	30	30	17.4 × 13.5	1,024 × 800	17 μm	~AUD$80K (imager only)
Sierra‐Olympic VayuHD	DJI M600	30	30	24 × 14.5	1,920 × 1,200	12 μm	~AUD$170K (imager only)

Note

The Jenoptik VarioCAM^®^ HD was used to evaluate whether rabbit warrens could be detected by a thermal imager. The FLIR Zenmuse XT640 and Sierra‐Olympic VayuHD were used to compare consumer products with high‐end professional products. The FLIR Zenmuse XT640 came as an integrated system with the DJI Inspire I drone. (Hz = frame rate).

### 2.3 Warren surveys

Determining which warren entrances belong to which warrens can be challenging in high‐density rabbit populations. For the purposes of this research, an entrance was part of the same warren if it was within 5 m of another entrance. When an entrance was detected that was more than 5 m away from another entrance, this was deemed to be part of a new warren. Single entrances that were >5 m away from other entrances were considered a single‐entrance warren. Warrens were regarded as active when one or more entrance had signs of use. This includes a lack of vegetation growing in the entrance, the presence of freshly excavated soil, fresh scat and/or the presence of rabbit footprints. Warrens where all entrances were covered in either debris (leaves and sticks), with cobwebs and with hard crusted soil were considered inactive. No further validation (e.g., excavation or trapping) was undertaken to confirm warren activity status.

All thermal imager surveys were conducted in the morning before first light to maximize the temperature differential between warren entrances and the surrounding terrain. All sites were visually inspected for rabbit warrens (active and inactive) on foot during the day (prior to the thermal survey), and all identified warrens were mapped with their GPS locations recorded. The ground and aerial surveys were independent, that is, the thermal imager transects were designed prior to visual inspection. In Part 1, we determined whether active rabbit warrens could be detected with a thermal imager. We flew the drone with the Jenoptik imager directly to the warren locations. In Part 2, we compared a professional imager (Vayu) to a consumer imager (Zenmuse). We established parallel flight transects to allow complete coverage of the area being investigated and to mimic the actual survey method that should be employed to search for warrens. We undertook visual counts of warrens and warren entrances in Part 2. Visual counts were undertaken upon arrival and before the drone flights. Parallel line transects approximately 10 m apart were walked, and all warrens and associated entrances were recorded. Once imagery from the drone flights was processed (see below), we undertook an additional visual inspection on foot to confirm entrances identified from the thermal imagery and to identify any false positives or negatives.

Prior to undertaking the surveys, we flew each imager at various flight heights and speeds to determine optimum picture quality. For the survey, the Zenmuse was flown at 3 m/s at 10 m above ground level (AGL). This resulted in a swath width of 15.6 m and a resolution of 1.4 pixels/cm. The Vayu was flown at 5 m/s at 40 m AGL resulting in a swath width of 39.8 m and a resolution of 2 pixels/cm. Transect spacing for each imager for the survey was determined by the swath width. Transects spacing for the Zenmuse was 11 m resulting in a transect overlap of 2.3 m either side of the image. Sixteen transects were required to cover the area taking two flights to complete. Transect spacing for the Vayu was 22 m resulting in a transect overlap of 8.5 m. Eight transects were required to cover the area which took one flight to complete. For both the Zenmuse and the Vayu, the imager was pointed 90 degrees to the horizontal during the surveys. During the surveys proper the drone was not stopped over entrances or warrens for confirmation of detection.

We downloaded the footage from the thermal imagers to an external hard drive and reviewed the footage from this drive using VLC media player 3.0.8. We recorded observations in a custom‐built Microsoft Excel (Microsoft Corporation, 2018) workbook which utilized the drone's tracklog to georeference observation locations. This file was then exported as a KML file and viewed in Google Earth Pro (Google Earth Pro, 2019) to aid in comparison between thermal imager and visual inspection detections.

Where transect imagery overlapped, double observations of warren entrances were removed from the worksheet before analysis. If a warren complex was identified on one transect, and additional warren entrances were identified on the immediate next transect in the same location, then a determination was made on whether these entrances belonged to the same warren or constituted a new warren. This ensured warren counts were not over‐estimated.

Warrens were classified by the amount of vegetation present that was likely to obscure entrances. Warrens with no vegetation present were classified as “open”, warrens obscured by vegetation (e.g., entrances were beneath shrubs) were classified as “vegetated” and warrens that had entrances in the open and obscured by vegetation were classified as “mixed”. These classifications also applied to the entrances associated with that warren for analysis (i.e., individual entrances in “mixed” warrens were not further classified into “open” or “vegetated” categories for analysis).

### 2.4 Statistical analysis

We used the *lme4* (Bates et al., 2015) and *lmerTest* (Kuznetsova et al., 2017) packages in R (R Core Team, 2019) to test for any difference in entrance count associated with imager. We used a mixed model with Poisson likelihood to account for the nested structure of imagers within warrens and the contrast of vegetation class between distinct warren sets. The package *emmeans* (Lenth, 2019) was used to inspect the mean entrance count under each vegetation and imager class. Additionally, we plotted difference between estimates versus average of the estimates to check for any patterning in case agreement depended on magnitude of observation as suggested by Altman and Bland (1983). To address any disagreement in terms of the presence or absence of entrances detected, the three pairings of methods (visual vs. Vayu, visual vs. Zenmuse and Vayu vs. Zenmuse) were examined by classifying entrance counts as equal to or greater than zero and forming two‐way tables (Table 2).

**TABLE 2:** Two‐way table used to quantify agreement (proportion of warrens where the imagers agree on presence or absence of warrens), False Nil (the proportion of warrens where imager “1” detected entrances but imager “2” detected zero entrances) and False Presence (the proportion of warrens where imager “1” detected zero entrances but imager “2” detected at least 1 entrance)

	Imager 1 Nil	Imager 1 Present
Imager 2 Nil	a	b
Imager 2 Present	c	d

Ratios of the table cells to the marginal totals can then be used to quantify:

**Agreement:** The proportion of warrens where the imagers agree on presence or absence of warrens.

$$\frac{a+d}{a+b+c+d}$$

**False Nil:** The proportion of warrens where imager “1” detected entrances but imager “2” detected zero entrances.

$$\frac{b}{b+d}$$

**False Presence:** The proportion of warrens where imager “1” detected zero entrances but imager “2” detected at least 1 entrance.

$$\frac{c}{a+c}$$

Note that the word “false” here is a value judgment given the arbitrary decision of which imager to designate as “1”. However, use of “visual” as the baseline to compare the performance of the thermal imagers seems justifiable. For all analysis, the significance level was set at 0.05.

## 3 RESULTS

### 3.1 Part 1 ‐ Detecting rabbit warrens

**FIGURE 1:**
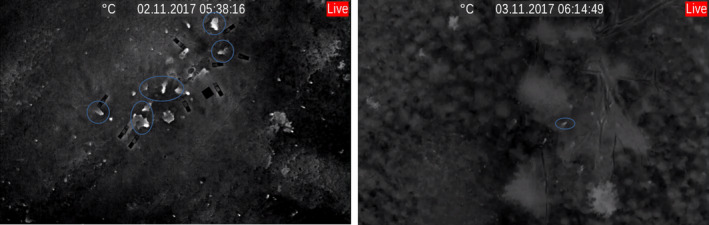
Active rabbit warrens detected by a thermal imager (Jenoptik VarioCAM^®^ HD) in (left) a high‐density area with little vegetation and (right) a low‐density area with extensive stands of serrated tussock and blackberry. Blue circles highlight some of the rabbit warren entrances. Rectangular objects (left image) are cage traps placed at warren entrances for another study; however, these cage traps provided confirmation that we were observing rabbit warren entrances

Active rabbit warrens were detected via thermal imagery in both high‐density and low‐density areas (Figure 1) using the Jenoptik. Rabbit warren entrances were detected under vegetation, including under blackberry (Figure 1) and where they were obscured by serrated tussock. No inactive rabbit warrens were detected by any of the thermal imagers during this study.

### 3.2 Part 2 ‐ Evaluation of imagers and visual inspections

**FIGURE 2:**
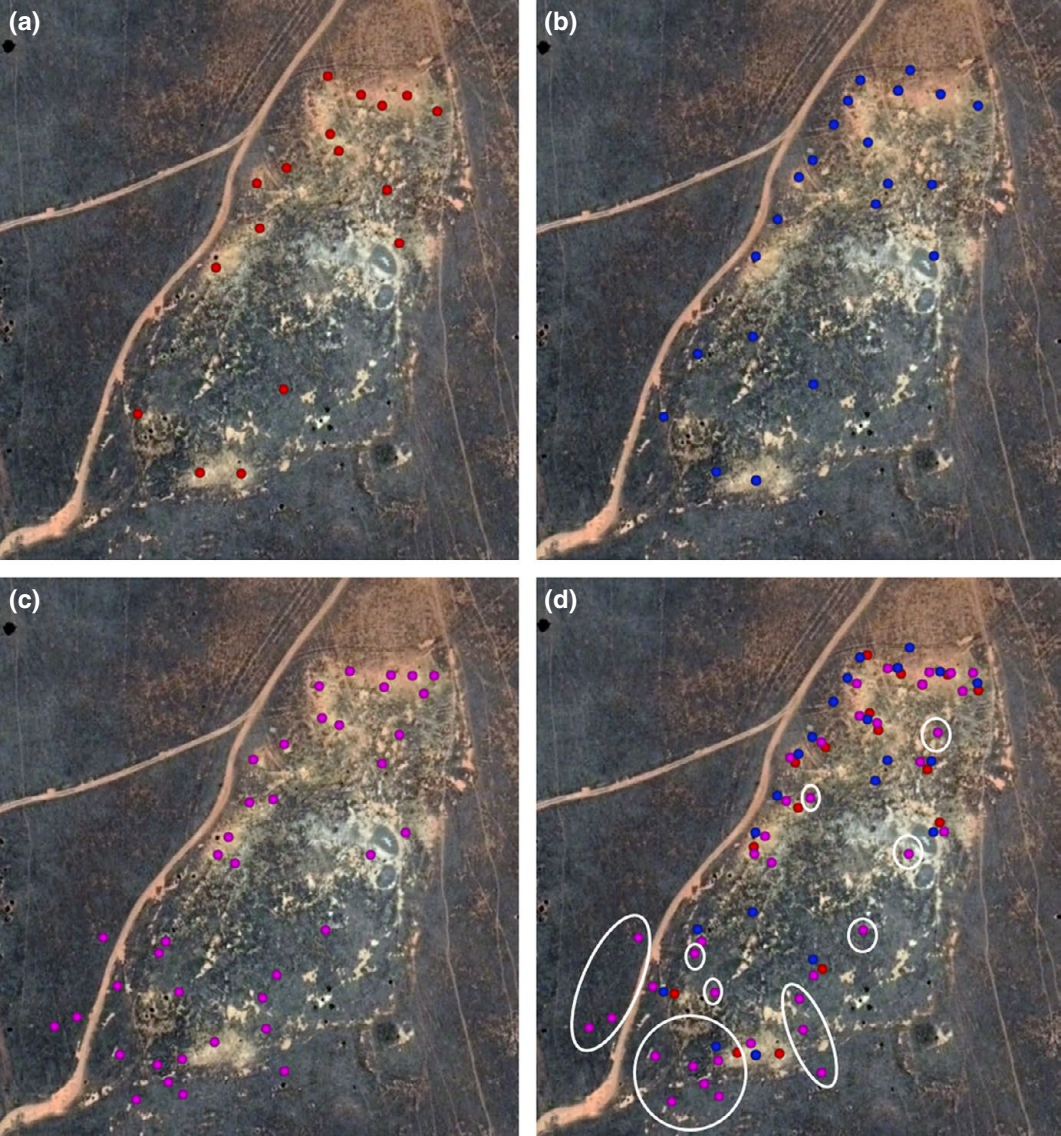
The locations of warrens detected by (a) visual assessment, (b) with the Vayu, (c) with the Zenmuse, and (d) a comparison of all detections from all three methods (with the false positives from the Zenmuse circled in white). The Zenmuse had a high rate of false‐positive imagery

**FIGURE 3:**
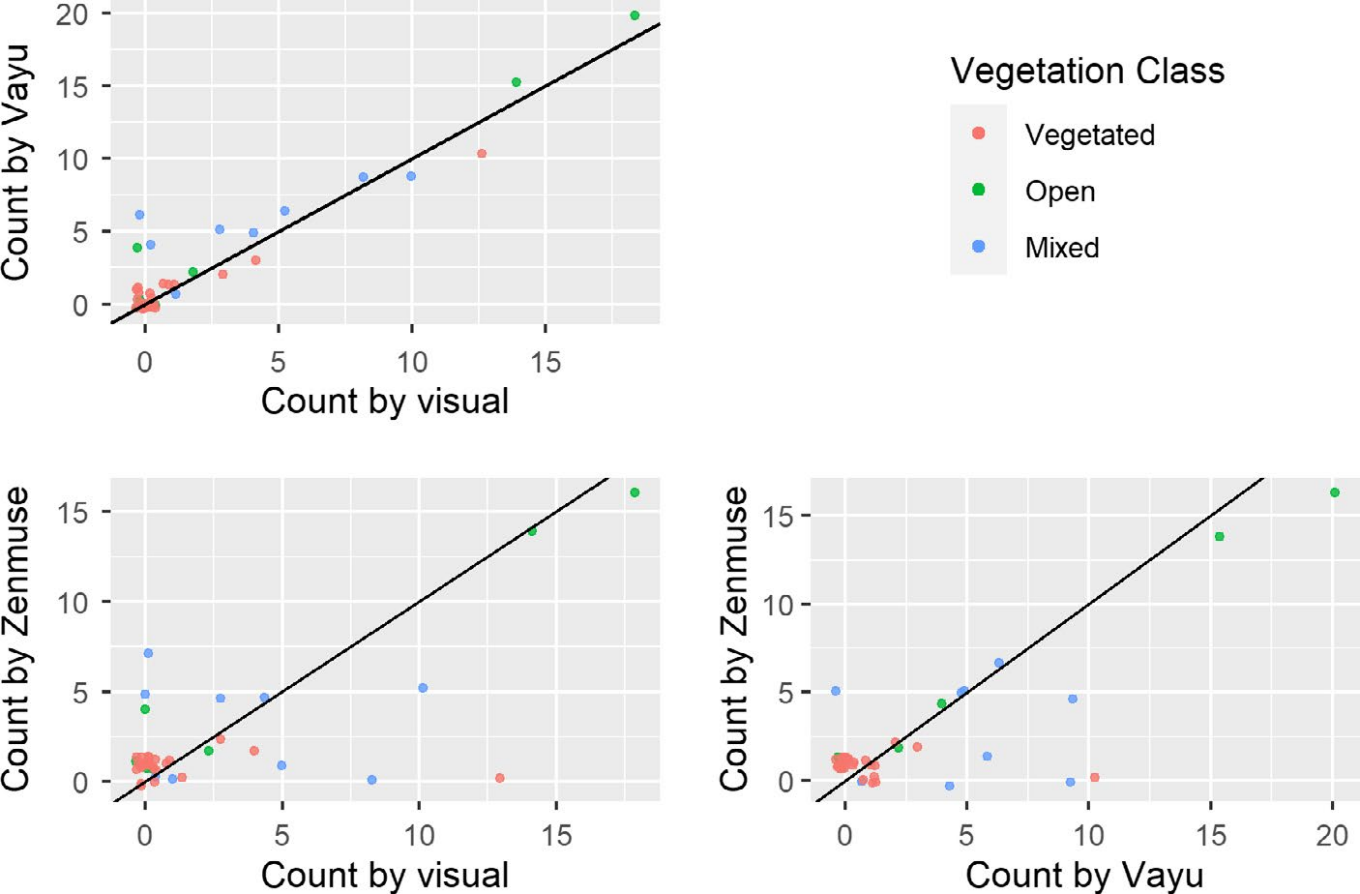
Pairs plots showing the correlation between counts under each imager or detection type (visual inspection, Vayu and Zenmuse) over all vegetation classes. 1:1 lines are added to show agreement and white noise added to each point in order to reveal overlapping points

Vegetation was a significant factor in the detection of rabbit warrens (*p *= <.001). There were 22 warrens present within the survey area. All warrens identified by visual assessment (*n* = 14) were identified in the imagery from the Vayu. Three warrens identified by visual assessment were not identified in the Zenmuse footage. However, both the Zenmuse and the Vayu detected more rabbit warrens and entrances than visual inspection (Table 3, Figure 2a). A pairs plot shows the correlation between counts under each imager over all vegetation classes (1:1 lines added to show agreement, Figure 3). The methods seem broadly in agreement.

**TABLE 3:** The number of warrens and entrances detected by each inspection method (Visual, Vayu, and Zenmuse) in each habitat type (O = open, *M* = mixed, V = vegetated)

Imager/Detection type	Number detected							
	Entrances				Warrens			
	O	M	V	Total	O	M	V	Total
Visual	34	31	22	**87**	3	6	5	**14**
Vayu	50	45	22	**117**	4	8	10	**22**
Zenmuse	39	28	22	**89**	7	6	20	**33**

Bold value is a tally of entrances and warrens detected by each method.

The Zenmuse detected significantly more entrances than both visual inspection and the Vayu (*p* =.049). Several entrances and warrens detected using the Zenmuse were later visually identified as false positives (*n* = 21 entrances, *n* = 19 warrens) (Figure 2c). The Vayu detected active warrens beneath vegetation and detected more warrens than visual inspection (Figure 2b, Table 3) with no false positives. Only 10 of the 22 warrens were detected by all three methods (Vayu, visual, and Zenmuse), and 14 of the 22 were detected by both the Vayu and visual inspection. Two warrens were detected by thermal imager (both types) that were not detected by visual inspection with an additional four warrens (three single and one four‐entrance warren) only detected by the Vayu (see Appendix S1).

Inspection of the mean entrance count under each vegetation and imager class (Table 4) revealed that, on average, both imager types and visual inspection detected almost the same number of entrances in open and vegetated areas. In mixed areas, the Vayu detected, on average, one more entrance per warren than visual inspection and up to twice as many entrances per warren than the Zenmuse.

**TABLE 4:** Mean entrance count for each imager/detection type under each vegetation type

Vegetation	Imager	Rate	SE	LCL	UCL
Open	Visual	2.027	0.897	0.851	4.828
	Vayu	2.445	1.068	1.038	5.757
	Zenmuse	2.325	1.019	0.985	5.491
Vegetated	Visual	0.528	0.164	0.287	0.973
	Vayu	0.505	0.159	0.273	0.936
	Zenmuse	0.505	0.159	0.273	0.936
Mixed	Visual	2.776	1.057	1.317	5.854
	Vayu	4.030	1.480	1.962	8.276
	Zenmuse	2.507	0.966	1.179	5.334

Note

Confidence level used: 0.95. Intervals are back transformed from the log scale.

Although the imagers were in agreement with respect to average entrance count over the survey, it is clear from Figure 3 that there is a degree of disagreement in terms of the presence or absence of entrances detected. Output from the contingency tables (Table 5) shows that the visual and Vayu had agreement on the presence or absence of entrances on 83% of warrens, noting that the Vayu detected entrances where the visual had not on 27% of warrens (Table 6). The Zenmuse was in poor agreement with the visual (27%) and Vayu (34%), and it seems the Zenmuse was particularly prone to detecting warrens that were “false positives” when compared to visual (85%) and Vayu (100%) (Table 6).

**TABLE 5:** Contingency tables for the three pairings of methods (visual vs. Vayu, visual vs. Zenmuse, and Vayu vs. Zenmuse) using classification of entrance counts as equal to or greater than zero

	Visual nil	Visual present
Vayu Nil	19	0
Vayu Present	7	15
Zenmuse Nil	4	4
Zenmuse Present	22	11

	Vayu nil	Vayu Present
Zenmuse Nil	0	8
Zenmuse Present	19	14

**TABLE 6:** Outputs from contingency tables for the three pairings of methods (visual vs. Vayu, visual vs. Zenmuse, and Vayu vs. Zenmuse) quantifying agreement (proportion of warrens where the imagers agree on presence or absence of warrens), False Nil (the proportion of warrens where imager “1” detected entrances but imager “2” detected zero entrances), and False Presence (The proportion of warrens where imager “1” detected zero entrances but imager “2” detected at least 1 entrance) between the methods

	*x*	*n*	Mean	LCL	UCL
Visual versus Vayu					
Agreement	34	41	0.83	0.68	0.93
False Nil	0	15	0.00	0.00	0.22
False Presence	7	26	0.27	0.12	0.48
Visual versus Zenmuse					
Agreement	15	41	0.37	0.22	0.53
False Nil	4	15	0.27	0.08	0.55
False Presence	22	26	0.85	0.65	0.96
Vayu versus Zenmuse					
Agreement	14	41	0.34	0.20	0.51
False Nil	8	22	0.36	0.17	0.59
False Presence	19	19	1.00	0.82	1.00

## 4 DISCUSSION

We believe this is the first study of its kind to show that thermal imagers can be used in systematic surveys to detect previously unknown burrows of fossorial animals. All three thermal imagers tested could detect active rabbit warrens. Both professional imagers could detect active rabbit warrens that were either obscured by, or under, vegetation, including blackberry bushes. The Vayu detected five more warrens in vegetated areas, three more warrens, and 14 more warren entrances in mixed vegetation and 16 more entrances in open areas than visual inspection. The Zenmuse initially appeared to detect more warrens than any other method, particularly in vegetated areas; however, inspection of these detections revealed a high number of false positives.

The Vayu, Zenmuse, and visual inspection detected the same number of entrances in vegetated habitat (Table 3), but the Vayu detected twice as many warrens, particularly single‐entrance warrens. Single entrance warrens may indicate a breeding stop or can be the start of a new warren. Either way, these single entrance warrens are important features to manage during a control program. Previous studies have reported difficulty in detecting animals with thermal imagery in areas of high canopy cover (Gooday et al. 2018; Mulero‐Pázmány et al., 2014), although not all studies report such issues (Witczuk et al., 2018, Lethbridge et al., 2019). While we were not focused on detecting the actual animal during this survey, high‐density rabbit populations emit a substantial amount of heat from warrens which was detected by the professional imagers, even when warrens were under vegetation. The professional imagers also had far superior exported imagery resulting in no false‐positive detections of rabbit warren entrances during postsurvey processing. We suspect that it is the exportable frame rate (Hz) that contributed to the poor performance of the Zenmuse, and potentially to the difficulty in detecting animals in vegetation in previous studies. For our study, the lower‐quality imagery made it difficult to distinguish between single warren entrances and other hot material such as rocks, which effectively looked like “hot blobs”. These “hot blobs” had little definition which made identification difficult. Similar issues have been reported previously (Gooday et al. 2018; Lethbridge et al., 2019). The viewing rate of the data stream from the imager (30 Hz) was enough to see rabbit warrens as the drone was flying, yet the exported video file at <9 Hz resulted in poor‐quality blurred imagery that was unsuitable for postsurvey analysis. Given that this technology is likely to be used to survey an area and have the imagery postprocessed and geotagged so that warrens can be mapped and subsequently removed, the lower export frame rate of <9 Hz of these imagers is insufficient for the task. These issues did not exist for the professional imagers which both had an export frame rate of 30 Hz. Consumers may be able to overcome the low export frame rate through the addition of an external high‐speed recorder to record the datastream from the imager at the viewing frame rate. This will add additional cost to the setup (AUD$1200‐1800), but this cost is insignificant compared to that of professional imagers.

Detecting active warrens and entrances gave no indication of the number of animals' present. The use of thermal imagers to estimate rabbit abundance in these scenarios is unreliable and not recommended. This technique provides presence data only. It is unknown how many rabbits are required to generate a heat signature at an entrance. Boonstra et al. (1994) and Hubbs et al. (2000) used thermal imaging to estimate the average number of hot entrances per arctic ground squirrel and then estimated abundance. Theoretically, the same should be possible for rabbits. However, factors such as warren size (number of entrances), warren depth, and even soil type are likely to influence the thermal signatures from entrances. Additionally, how many rabbits are required to generate a heat signature in a variety of these conditions needs to be understood. Further research should include the removal of all rabbits from warrens of varying depths in varying soil types to determine the minimum number of rabbits required to emit a detectable heat signature.

Thermal imaging technology is becoming more widely available but is still a costly technique. However, the cost of missing warren entrances in a ripping program may be greater. The opportunity for rabbits to reopen warrens through missed entrances has the potential to negate tens‐of‐thousands of dollars of work on a local scale and millions of dollars on the national scale. Australians spend approximately AUD$6 million per year on rabbit control programs (Gong et al., 2009), and many programs include warren ripping. Warren ripping can cost anywhere from AUD$50‐$250/Ha, depending on the size of the equipment used, the level of infestation and the soil type (and therefore warren depth). For our survey area in western NSW, we estimated that ripping would cost AUD$250/Ha given the very high rabbit population and an average warren depth of 1.5–2.5 m (resulting in the need for larger equipment to ensure an effective ripping program). This results in a ripping cost of AUD$1,527.50 for this 6.11 Ha. Ripping programs tend not to happen in isolation and are often part of a multitool approach with associated poisoning programs (approx. AUD$50/Ha) at a minimum. This brings the cost of initial control for these 6.11 Ha to AUD$1,833. Rabbits from surrounding areas can quickly reinvade and repopulate these 6.11 Ha if all the warrens and entrances are not detected (McPhee & Butler, 2010). The professional imager detected 30 more entrances and eight more warrens overall than visual inspection or the corrected consumer imager. If warren ripping was undertaken at this site using the visual or corrected consumer imager data alone, then up to eight warrens could have been missed, rendering the control program ineffective. Repeated across warrens on average small holdings (30–100 Ha), the cost of missed warrens/entrances and having to repeat control programs soon becomes considerable.

While this research focuses on the detection of active rabbit warrens and their entrances, the inadequacies of the exported imagery from the consumer imager will be important in other areas of thermal research. We expect that professional‐grade thermal imagers will not be widely used in many wildlife research projects simply due to their cost. However, as consumer‐grade thermal imaging equipment becomes increasingly available, there is an opportunity to incorporate thermal imagery more cost‐effectively into ecology research projects. More information needs to be gathered on how these consumer‐grade thermal imagers perform in detecting a range of wildlife species. Specifically, how the low exportable frame rate affects postflight image processing and the occurrence of “hot blobs” and species identification. This will become increasingly important as the field moves toward the use of automated detection algorithms in footage review.

## 5 CONCLUSIONS

Thermal imaging technology provides an efficient method for detecting rabbit warrens and entrances in all vegetation types (open, vegetated, and mixed), surpassing visual inspection alone. Improved detection of warrens and their entrances can lead to more effective control programs, ensuring all warrens and entrances within an area are identified. This should lead to reduced control costs over time due to decreased rates of reopening. Both consumer and professional thermal imagers can be used; however, consumer imagers should be supplemented with additional technology due to their poor exportable frame rate. Low exportable frame rates produce noisy and blurred imagery. This causes hot spots to look like indistinguishable white blobs which ultimately results in a high number of false‐positive detections.

## CONFLICT OF INTEREST

None declared.

## AUTHOR CONTRIBUTIONS

**Tarnya Cox:** Conceptualization (lead); data curation (lead); formal analysis (supporting); funding acquisition (lead); methodology (lead); project administration (lead); writing–original draft (lead); writing–review and editing (lead). **Robert Matthews:** Funding acquisition (supporting); investigation (equal); methodology (equal); project administration (supporting); writing–original draft (supporting); writing–review and editing (supporting). **Grant Halverson:** Conceptualization (equal); data curation (supporting); funding acquisition (equal); investigation (equal); methodology (equal); project administration (supporting). **Stephen Morris:** Formal analysis (lead); writing–original draft (equal).

## Supporting information

Appendix S1

## Data Availability

The data used in the analysis is available in Appendix S1 and available at Dryad: https://doi.org/10.5061/dryad.cz8w9gj33. The original thermal footage is the property of the New South Wales Department of Primary Industries. To discuss access please email the corresponding author.
