# Supplementary material for: Hot stuff in the bushes: Thermal imagers and the detection of burrows in vegetated sites
Source: Ecol Evol. 2021 Apr 1;11(11):6406–14. doi: 10.1002/ece3.7491 (PMC8207428; doi:10.1002/ece3.7491)
Supplement: Supplementary file 1 — Appendix S1 [file ECE3-11-6406-s001.docx]

Appendix

List of warrens, vegetation type and number of warren entrances detected by each detection method/imager type. Grey rows indicate false positive detections from the Zenmuse footage.

|  |  | Number of entrances detected | | | |
| --- | --- | --- | --- | --- | --- |
| vegetation | warren | visual | vayu | zenmuse |  |
| v | A | 0 | 0 | 1 |  |
| v | B | 0 | 0 | 1 |  |
| v | C | 0 | 0 | 1 |  |
| m | D | 1 | 1 | 0 |  |
| m | E | 0 | 6 | 7 |  |
| v | F | 0 | 1 | 0 |  |
| v | G | 3 | 2 | 2 |  |
| m | H | 4 | 5 | 5 |  |
| m | I | 10 | 9 | 5 |  |
| o | J | 18 | 20 | 16 |  |
| v | K | 0 | 1 | 1 |  |
| v | L | 0 | 0 | 1 |  |
| v | M | 4 | 3 | 2 |  |
| v | N | 0 | 0 | 1 |  |
| v | O | 0 | 0 | 1 |  |
| v | P | 0 | 1 | 0 |  |
| o | Q | 0 | 0 | 1 |  |
| o | R | 14 | 15 | 14 |  |
| o | S | 2 | 2 | 2 |  |
| m | T | 0 | 4 | 0 |  |
| m | U | 8 | 9 | 0 |  |
| v | V | 1 | 1 | 0 |  |
| v | W | 0 | 1 | 0 |  |
| v | X | 13 | 10 | 0 |  |
| v | Y | 0 | 0 | 1 |  |
| v | Z | 0 | 0 | 1 |  |
| v | AA | 0 | 0 | 1 |  |
| v | AB | 0 | 0 | 1 |  |
| v | AC | 0 | 0 | 1 |  |
| o | AD | 0 | 4 | 4 |  |
| v | AE | 1 | 1 | 1 |  |
| v | AF | 0 | 0 | 1 |  |
| v | AG | 1 | 1 | 1 |  |
| m | AH | 0 | 0 | 5 |  |
| o | AI | 0 | 0 | 1 |  |
| m | AJ | 3 | 5 | 5 |  |
| m | AK | 5 | 6 | 1 |  |
| o | AL | 0 | 0 | 1 |  |
| v | AM | 0 | 0 | 1 |  |
| v | AN | 0 | 0 | 1 |  |
| v | AO | 0 | 0 | 1 |  |
